# Reduced Expression of the Vesicular Acetylcholine Transporter and Neurotransmitter Content Affects Synaptic Vesicle Distribution and Shape in Mouse Neuromuscular Junction

**DOI:** 10.1371/journal.pone.0078342

**Published:** 2013-11-08

**Authors:** Hermann A. Rodrigues, Matheus de C. Fonseca, Wallace L. Camargo, Patrícia M. A. Lima, Patrícia M. Martinelli, Lígia A. Naves, Vânia F. Prado, Marco A. M. Prado, Cristina Guatimosim

**Affiliations:** 1 Departamento de Morfologia, ICB, Universidade Federal de Minas Gerais, Belo Horizonte, Brasil; 2 Departamento de Fisiologia e Biofísica, ICB, Universidade Federal de Minas Gerais, Belo Horizonte, Brasil; 3 Departamento de Engenharia de Biossistemas, Universidade Federal de São João Del Rei, São João Del Rei, Brasil; 4 Robarts Research Institute and Department of Physiology and Pharmacology and Anatomy & Cell Biology, University of Western Ontario, London, ON, Canada; University of Sydney, Australia

## Abstract

In vertebrates, nerve muscle communication is mediated by the release of the neurotransmitter acetylcholine packed inside synaptic vesicles by a specific vesicular acetylcholine transporter (VAChT). Here we used a mouse model (VAChT KD^HOM^) with 70% reduction in the expression of VAChT to investigate the morphological and functional consequences of a decreased acetylcholine uptake and release in neuromuscular synapses. Upon hypertonic stimulation, VAChT KD^HOM^ mice presented a reduction in the amplitude and frequency of miniature endplate potentials, FM 1–43 staining intensity, total number of synaptic vesicles and altered distribution of vesicles within the synaptic terminal. In contrast, under electrical stimulation or no stimulation, VAChT KD^HOM^ neuromuscular junctions did not differ from WT on total number of vesicles but showed altered distribution. Additionally, motor nerve terminals in VAChT KD^HOM^ exhibited small and flattened synaptic vesicles similar to that observed in WT mice treated with vesamicol that blocks acetylcholine uptake. Based on these results, we propose that decreased VAChT levels affect synaptic vesicle biogenesis and distribution whereas a lower ACh content affects vesicles shape.

## Introduction

Acetylcholine (ACh) plays an important role during nervous system development [1], [2], [3]. In mammalian neuromuscular junction (NMJ), ACh is synthesized in presynaptic terminals of cholinergic neurons from choline and acetyl-coenzyme A (acetyl-CoA) by choline acetyltransferase (ChAT) and then transported into synaptic vesicles (SVs) by the vesicular acetylcholine transporter (VAChT) [4]. After depolarization, ACh is released into the synaptic cleft and binds to nicotinic receptors present on the postsynaptic muscle membrane, transmitting the signal for muscular contraction [4], [5].

The release of neurotransmitters depends on its storage into SVs [6], [7], [8], and VAChT expression represents a key point in the regulation of cholinergic transmission [9], [10]. VAChT knockout (VAChT^del/del^) mice appear to have normal SV recycling, but they are unable to store or release sufficient ACh in response to neural activity. As a consequence, they do not survive more than few minutes after birth [3]. In contrast, mice with 70% reduced VAChT expression (VAChT KD^HOM^) reach adulthood, but these animals show cardiac dysfunction and cognitive alterations [3], [9], [11]. In addition, VAChT KD^HOM^ mice present a pronounced deficit in neuromuscular transmission characterized by a reduction in quantal content and size, reduced miniature end-plate potentials frequency, impairment of motor performance and severe deficit in muscle strength [9], [10]. Understanding how synaptic terminals respond to reduced expression of this transporter is relevant, as decreased levels of VAChT have been reported in response to drug treatments [12], [13], as well as in distinct neurodegenerative diseases [14], [15]. To investigate whether decreased levels of VAChT, and consequently reduced ACh storage, can regulate any aspect of the SV cycle, studies using the NMJ are ideal, due to the homogenous cholinergic nature of this synapse and its accessibility to imaging and electron microscopy.

Although studies using the fluorescent dye FM1-43 suggested that VAChT KD^HOM^ mice appear to have normal SV cycle [9], a detailed ultrastructural investigation of the NMJ in these mice was not performed. In the present study we characterized, at the ultrastructure level, the morphology of synaptic nerve terminals from diaphragm muscles of VAChT KD^HOM^ mice. Our data show that reduced expression of VAChT does not interfere with the overall morphology of the NMJ, but changes the distribution of SV within the nerve terminal. In addition, reduced expression of VAChT changes the shape of SVs suggesting that neurotransmitter content may play a key role in maintaining their structure. Our results demonstrate a link between ACh storage and regulation of SV recycling.

## Materials and Methods

### Drugs and chemicals

FM1-43fx and ProLong® Gold antifade were purchased from Invitrogen^TM^; d-tubocurarine, ADVASEP-7, (±)-Vesamicol hydrochloride were purchased from Sigma-Aldrich and µ-conotoxin was obtained from Alomone Labs. All other chemical and reagents were of analytical grade.

### Ethics Statement

All experimental procedures were carried out in accordance with protocol approved by the local animal care committee (CETEA-UFMG – protocol 40/2009) and followed NIH guidelines for the Care and Use of Animals in Research and Teaching.

### Nerve-muscle preparation

Generation of VAChT KD^HOM^ mice has been previously described in detail [9]. The experiments were performed using adult 3 month-old VAChT WT and VAChT KD^HOM^ mice. The diaphragm muscle associated with the corresponding nerve were dissected out, split in two hemidiaphragms and bathed in mouse Ringer solution (135 mM NaCl, 5 mM KCl, 2 mM CaCl_2_, 1 mM MgCl_2_, 12 mM NaHCO_3_, 1 mM NaH_2_PO_4_, 11 mM D-glucose, pH 7.4) and bubbled with a mixture of 5%CO2/95%O2. In transmission electron microscopy experiments, diaphragm muscles were fixed in ice-cold modified Karnovsky solution fixative (4.0% paraformaldehyde and 2.5% glutaraldehyde in 0.1 M sodium cacodylate buffer).

### Monitoring endocytosis with FM1–43fx

Experiments with FM1-43 were performed according to the protocol previously described [16], [17] except that a fixable (fx) FM1-43 analog was used. Diaphragm muscles were stimulated with hypertonic sucrose solution (500 mM) containing FM1-43fx (8 μM) for 10 min. After stimulation, the preparation was maintained at rest in normal Ringer solution with FM1-43fx for 10 min to guarantee maximal FM1-43fx uptake during compensatory endocytosis. Following labeling, muscles were washed for 1 hour in normal mouse Ringer containing Advasep-7 (1 mM) to remove extracellular FM1-43fx. For labeling of nicotinic acetylcholine receptor (nAChR) clusters, the preparations were exposed to α-bungarotoxin-Alexa 594 (12 µM) during 20 minutes and then washed [16]. Diaphragms were post-fixed with paraformaldehyde 4% in PBS for 40 min and mounted onto glass slide using ProLong® Gold antifade reagent.

### Confocal microscopy and image analysis

Images of NMJs stained with FM1-43fx and α-bungarotoxin were acquired using a 40x oil immersion (NA 1.30) objective attached to a laser-scanning confocal microscope (Zeiss 510 META) located at Center of Acquisition and Processing of Images (CAPI) – ICB – UFMG. An argon (488 nm) and helium-neon (543 nm) laser were used for excitation of terminals stained with FM 1–43fx and nAChR cluster marked with α-bungarotoxin, respectively. Z series optical sections were collected at 2.0 µm intervals and the whole hemidiaphragms were scanned. The nerve terminals were indentified considering their colocalization with nAChR clusters. Images were converted to gray scale format (8 bits) and each synaptic element was individually evaluated and the mean fluorescence intensity was considered for comparison between genotypes.

### Electrophysiological recordings

Standard intracellular recording techniques were used to record miniature endplate potentials (MEPPs) with an Axopatch-200 amplifier (Molecular Devices). Recordings were low-pass filtered at 5 KHz and amplified 50X prior to digitization and acquisition on a computer running WinEDR (John Dempster, University of Strathclyde). Microelectrodes were fabricated from borosilicate glass and had resistances of 8–15 MΩ when filled with 3 M KCl. MEPPs were recorded during 10 min in presence of normal Ringer and during exposure to sucrose hypertonic solution (500 mM). μ-Conotoxin GIIIB (0.37 μM) was added to avoid muscle contraction. MEPP amplitudes were recorded and scaled for differences in resting potential using −70 mV as the standard. MEPPs were recorded in the same fiber for 10 min before and during application of hypertonic sucrose.

### Transmission Electron Microscopy (TEM)

For ultrastructural characterization, VAChT WT and VAChT KD^HOM^ mice were anesthetized with ketamine/xilazine (70/10 mg/kg) i.p. and transcardially perfused with ice-cold PBS for 10 min, followed by ice-cold fixative modified Karnovsky solution for 10 min. Perfused diaphragm muscles were maintained in fixative solution overnight at 4°C. For experiments with stimulation, nerve muscle preparations were electrically stimulated (20 Hz/5 min) through the phrenic nerve (calcium-dependent stimuli) and immediately fixed or stimulated with hypertonic sucrose solution (500 mM) for 10 min (calcium-independent stimuli). After stimulation, the preparation was maintained at rest for 10 minutes in mouse Ringer solution without sucrose and fixed in ice-cold modified Karnovsky solution overnight at 4°C.

To investigate the effects of reduced ACh storage in SVs morphology, the diaphragm muscle from C57BL/6 mice was electrically stimulated (3 Hz/20 min) through the phrenic nerve in the presence of (±)-vesamicol (4 μM), a VAChT inhibitor [18] and immediately fixed overnight at 4°C.

After fixation, samples were washed with cacodylate buffer (0.1 M), cut into several pieces, post-fixed in reduced osmium (1% osmium tetroxide containing 1,6% potassium ferrocyanide), contrasted *en bloc* with uranyl acetate (2% uranyl acetate in deionized water), dehydrated through an ascending series of ethanol solutions and embedded in EPON. Blocks were sectioned (50 nm) and collected on 200 or 300 mesh copper grids and contrasted with lead citrate. Serial ultrathin sections (50 nm) were collected and mounted on formvar-coated slot cooper grids and contrasted with lead citrate. Sections were viewed with a Tecnai-G2-Spirit-FEI/Quanta electron microscope (120 kV Philips) located at Microscopy Center – UFMG or with an EM 10 Zeiss electron microscope (80 Kv) located at CAPI (ICB – UFMG).

### TEM image analysis

NMJs of interest were selected based on the presence of junctional folds in the postsynaptic membrane. Single sections through NMJs of interest were traced and the terminal areas (cross section area of each nerve terminal), postsynaptic junctional folds length and SV number were determined. SV distribution was evaluated by quantification of the vesicles located at different distances from the active zone within the selected area (small and big circle), as previously described [19], [20] and vesicles counted were marked to prevent their recounting. Vesicles within 50 to 300 nm of the presynaptic membrane were counted in 50 nm bins. We have defined active zone as presynaptic regions immediately opposed to postsynaptic fold within 300 nms from the plasma membrane. Vesicle circumference was measured using the equation 2π [(d_1_^2^+d_2_^2^)/2]^0.5^ considering the longest diameter (*d*1) and the diameter at right angles (*d*2) [8]. SVs shape was determined using the equation: shape factor  =  (4× π × area)/(perimeter)^2^. This parameter reaches a maximum of 1 for a circular object [21]. All image analysis in this study was performed “blind” in the sense that the person performing the analysis did not know what genotype or treatment the sample had received.

### Statistical Analysis

Image analysis was performed using the program Image J (Wayne Rasband, National Institutes of Health, USA) or Image-Pro Plus® 4.0 (4.5 (Media Cybernetics, Silver Spring, MD, EUA) or AxioVision 4.8 (Carl Zeiss). Data were analyzed in Microsoft Excel and plotted using the program SigmaPlot 10.0 (SyStat Software) or GraphPad Prism 4 or Igor (Wavemetrics). The averages ± standard error of the mean (SEM) from each group were calculated and compared. Statistical significance was evaluated using the paired or un-paired Student's *t*-test or the Komogorov-Smirnov test, as described in the text. Values of P<0.05 were considered significant.

## Results

**Figure 1:**
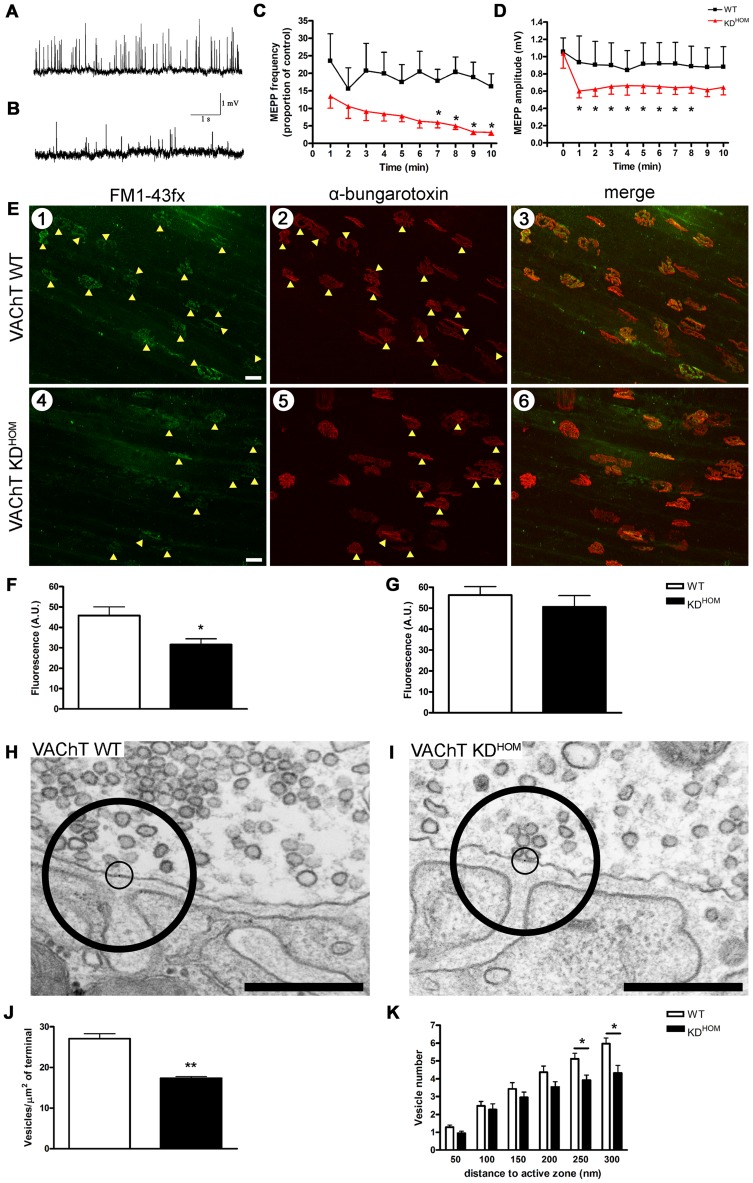
Alteration in SVs recycling and distribution after hypertonic sucrose stimulation in VAChT KD^HOM^ NMJs. A and B – Representative records of MEPPs obtained from the diaphragm muscle of VAChT WT and VAChT KD^HOM^ mice, respectively, measured in the presence of hypertonic sucrose solution (500 mM) at the end of 10 minutes. C -Graph comparing the mean values of normalized MEPPs frequency measured in the presence of hypertonic sucrose during 10 minutes. The results were normalized using the basal MEPPs values for each genotype. D – Graph showing the mean values of MEPP amplitude before (time zero) and during 10 minutes in hypertonic solution. In C and D all results are expressed as mean ± SEM. * p<0.05; n = 4 animals per genotype E– Confocal representative images of NMJs from the diaphragm muscle of VAChT WT (E1–E3) and VAChT KD^HOM^ mice (E4–E6): E1 and E4– presynaptic terminals stained with FM1-43 fx after hypertonic stimulation for 10 min; E2 and E5– postsynaptic nAChR clusters stained with α-bungarotoxin-Alexa 594; E3 and E6– colocalization of synaptic elements. Scale bar  = 10 μm. F– Graph showing the fluorescence intensity of the presynaptic terminal in arbitrary units (A.U.) (* p<0.05). G– Graph comparing the fluorescence intensity of the postsynaptic nAChR clusters in arbitrary units (A.U.). (n = 3 animals of each genotype). H and I– Representative electron-micrographs of two diaphragm NMJs profiles of VAChT WT and VAChT KD^HOM^ mice after hypertonic stimulation for 10 min, showing altered distribution and reduced number of SVs inside the areas labeled within the circles: 50 and 300 nm from the membrane, small and big circles respectively. Scale bar  = 500 nm. Magnification 50.000x. J– Graph comparing the relationship of SVs/μm^2^ of presynaptic terminal. (** p<0.01). K– Graph showing the average number of SVs located at different distances from the presynaptic active zones. (n = 3 individual animals per genotype; * p<0.05).

Previous studies from our research group showed that internalization of FM1-43 by motor terminals of VAChT KD^HOM^ mice and WT controls in response to electrical stimulation is very similar, suggesting that endocytosis is not affected in VAChT KD^HOM^ mice [9]. Likewise, internalization of FM1-43 by NMJs of VAChT^del/del^ mice indicates the existence of bulk SV recycling even in the absence of this transporter [3]. To further investigate the recycling and distribution of SV from the readily releasable pool (RRP) in nerve terminals from diaphragm muscle of VAChT KD^HOM^ mice we used hypertonic sucrose (500 mM) as a stimulus [22]. Hypertonic extracellular solution has been shown to increase the frequency of MEPPs at the frog and rat NMJs [23], [24], [25], [26]. The mechanism behind this increase is still unknown, however, it has been described that hypertonicity does not require Ca^2+^ influx or release from internal stores and may facilitate fusion of docked vesicles [22], [27]. Figures 1A and 1B show two representative traces of MEPPs measured from diaphragm neuromuscular preparations of VAChT WT and KD^HOM^, respectively, at the end of 10 minutes in the presence of hypertonic sucrose solution (500 mM). Before hypertonic solution, MEPPs frequencies were: VAChT WT (0.4±0.1 s^−1^) and VAChT KD^HOM^ (0.7±0.1 s^−1^) (mean ± SEM). Application of hypertonic solution increased MEPPs frequency in both WT and VAChT KD^HOM^ preparations. In WT, the increased frequency was sustained for ten minutes. In contrast, MEPPs frequency in VAChT KD^HOM^ decreased steadily from the peak. After 10 minutes of hypertonic stimulation, the MEPPs frequency in VAChT WT was 16.±3.7 times the pre-stimulation frequency whereas in VAChT KD^HOM^ frequency was only 3.1±0.8 times the pre-stimulation value (Figure 1C – p<0.05; unpaired Student's *t*-test; 4 muscle fiber for each genotype). Before hypertonic solution, MEPP amplitude was: VAChT WT (1.1±0.2 mV) VAChT KD^HOM^ (1.0±0.2 mV) (mean ± SEM). Application of hypertonic solution decreased MEPP amplitude in the mutants but not in WT. The decrease in amplitude in VAChT KD^HOM^ was seen as soon as the first minute, where MEPP amplitude was 0.6±0.1 mV (Figure 1D – p<0.05; paired Student's *t*-test; 4 muscle fiber for each genotype). These data suggest that during hypertonic stimulation, vesicle filling cannot keep up with release and VAChT KD^HOM^ mutants release partially filled vesicles. The decrease in frequency, which occurs later, may reflect either reduced release or release of empty vesicles. If the latter, it suggests that vesicles filling occurs in at least two stages.

One potential mechanism to explain these results is that some synaptic vesicles in the RRP of VAChT KD^HOM^ mice have low levels of neurotransmitter that make them invisible for electrophysiology recordings. A second potential mechanism is that in the absence of VAChT, a population of SVs in the RRP is impaired. To determine which of these two potential mechanisms are involved with reduced MEPP frequency in VAChT KD^HOM^ mice in response to hypertonic stimulation, we initially measured internalization of FM1-43fx to evaluate endocytosis under this condition. Figures 1E1 and E4 show representative images of diaphragm nerve terminals labeled with FM1-43 fx from VAChT WT and VAChT KD^HOM^ mice, respectively. When we measured fluorescence intensity, we observed that the presynaptic terminals of VAChT KD^HOM^ showed decreased fluorescent signal when compared to terminals from VAChT WT mice [WT  = 45.87±4.190 A.U. (mean ± SEM); KD^HOM^ = 31.60±2.809 A.U.; p<0.05; unpaired Student's *t*-test], suggesting that recycling of SVs of the RRP in VAChT KD^HOM^ might be reduced (Figure 1F – quantification of 1248 and 572 presynaptic nerve terminal in WT and KD^HOM^, respectively; n = 3 mice per genotype). Because hypertonic stimuli recruit a small number of SVs, FM1-43 fx internalization and fluorescence levels of presynaptic terminals are reduced in both genotypes. So to ensure that the measurement of fluorescent signal was really occurring at the nerve terminals level we performed the labeling of postsynaptic nAChR clusters with α-bungarotoxin to identify the precise location of the presynaptic terminals. Figures 1E2 and E5 show representative images of diaphragm postsynaptic nAChR clusters labeled with α-bungarotoxin-Alexa 594 from VAChT WT and VAChT KD^HOM^ mice, respectively. We observed that fluorescence intensity of postsynaptic elements was similar between genotypes [WT  = 56.21±4.088 A.U. (mean ± SEM); KD^HOM^ = 50.67±5.285 A.U.; p = 0.4535; unpaired Student's *t*-test] (Figure 1G –quantification of 1814 and 1609 postsynaptic nAChR clusters in WT and KD^HOM^, respectively; n = 3 mice per genotype). Figures 1E3 and E6 show the colocalization of pre and postsynaptic elements in diaphragm muscle from VAChT WT and VAChT KD^HOM^ mice, respectively.

To precisely determine whether the NMJ of VAChT KD^HOM^ mice show reduction in the number of SVs from RRP when submitted to hypertonic stimulation, we used transmission electron microscopy. Ultrastructural analysis showed a reduction in the total number and altered distribution of SVs in presynaptic nerve terminals from VAChT KD^HOM^ animals compared to WT (Figure 1H and 1I – small and big circles standing for synaptic vesicles located within 50 and 300 nm from the plasma membrane respectively). Morphometric analysis confirmed that the total number of SVs/μm^2^ was significantly reduced in VAChT KD^HOM^ mice (17.0±0.0 SVs) when compared to WT controls (27.0±1.0 SVs) (Figure 1J – p<0.01, unpaired Student's *t*-test). Additionally, we analyzed the distribution of SVs in motor nerve terminals of VAChT KD^HOM^ mice after sucrose stimulation and found a altered distribution of SVs located near the presynaptic active zones when compared with VAChT WT mice (Figure 1K – 250 nm: WT  = 5.0 SVs (mean), KD^HOM^  = 4.0 SVs; 300 nm: WT  = 6.0 SVs, KD^HOM^  = 4.0 SVs; p<0.05, unpaired Student's *t*-test; 15 nerve terminal profiles per genotype; n = 3 mice per condition).

**Figure 2:**
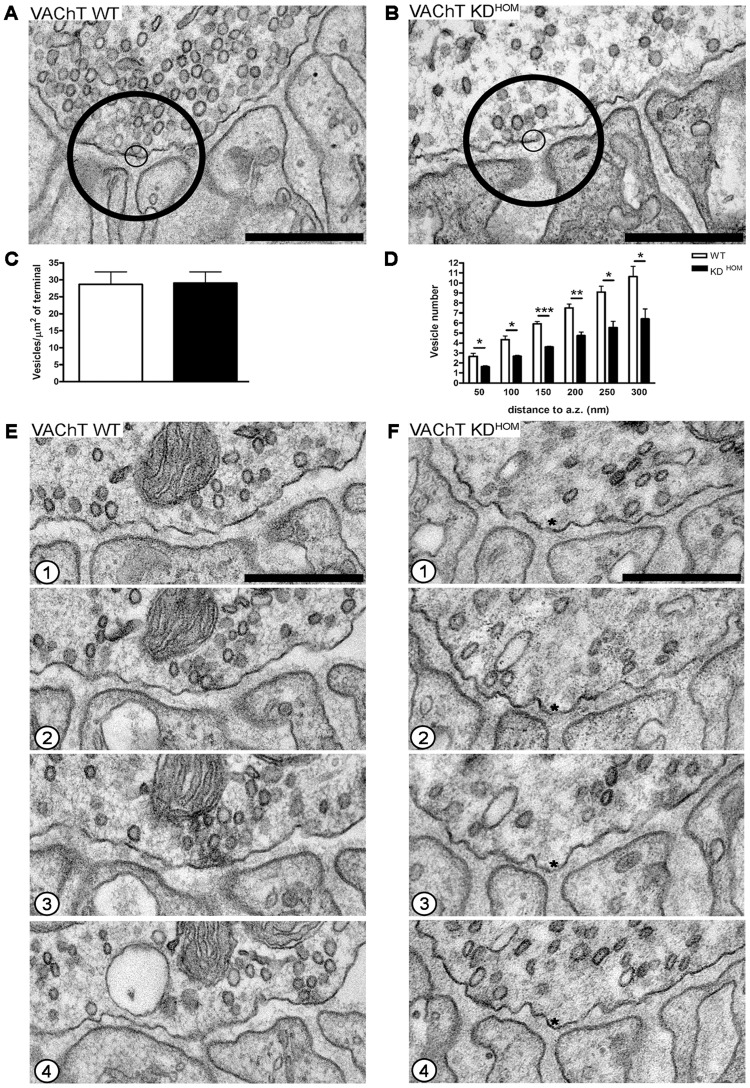
The reduced expression of VAChT alters SVs distribution involved in eletrically stimulated NMJs. A and B – Representative images of two NMJs from diaphragm muscle of VAChT WT and VAChT KD^HOM^ mice after electrical stimulation (20 Hz for 5 minutes) showing an altered SVs distribution from the active zone within the circles: 50 and 300 nm from the membrane, small and big circles respectively. Scale bar  = 500 nm. Magnification 50.000x. C– Graph of the ratio SVs/area of presynaptic terminal in μm^2^. D – Graph showing the average number of SVs located at different distances from the presynaptic active zones. E and F– Four serial sections of NMJs from VAChT WT (E1–E4) and VAChT KD^HOM^ (F1–F4) diaphragm showing the altered SVs distribution in the active zone (* represent areas depleted of SVs touching the membrane) of motor terminals of VAChT KD^HOM^ after electrical stimulation. Scale bar  = 500 nm. Magnification 50.000x. (n = 3 individual animals per genotype. * p<0.05, ** p = 0.005; *** p = 0.0006).

We next investigated at the EM level, the distribution and recycling of SVs in diaphragm nerve terminals of VAChT KD^HOM^ mice after electrical stimulation (20 Hz/5 min). We observed an altered distribution of SVs near the presynaptic active zones from NMJs of VAChT KD^HOM^ (Figure 2A and 2B – small and big circles standing for synaptic vesicles located within 50 and 300 nm from the plasma membrane respectively). However, we did not observe any difference in the total number of SVs/μm^2^ of terminal between genotypes (Figure 2C – WT  = 29.0±4.0 SVs [mean ± SEM]; KD^HOM^  = 29.0±3.0 SVs; p>0.05; unpaired Student's *t*-test; 15 nerve terminals profile per genotype; n = 3 mice per genotype), confirming our previous observation that SV recycling evoked by electrical stimulation is normal in VAChT KD^HOM^ nerve terminals [9]. Quantitative analysis confirmed that the NMJs of VAChT KD^HOM^ mice exhibited an altered distribution of SVs located at different distances from presynaptic active zone after electrical stimulation when compared with the VAChT WT mice [Figure 2D – 50 nm: WT  = 3.0 SVs (mean), KD^HOM^  = 2.0 SVs; 100 nm: WT  = 4.0 SVs, KD^HOM^  = 3.0 SVs; 150 nm: WT  = 6.0 SVs, KD^HOM^  = 4.0 SVs; 200 nm: WT  = 8.0 SVs, KD^HOM^  = 5.0 SVs; 250 nm: WT  = 9.0 SVs, KD^HOM^  = 6.0 SVs; 300 nm: WT  = 11.0 SVs, KD^HOM^  = 6.0 SVs; p<0.05, unpaired Student's *t*-test; we analyzed 15 nerve terminals profiles per genotype; n = 3 mice per genotype]. Figures 2E and 2F show four serial sections (50 nm thick) of NMJs of VAChT WT (E1–E4) and VAChT KD^HOM^ (F1–F4) mice after electrical stimulation (20 Hz/5 min), respectively. These serial sections of NMJs of VAChT KD^HOM^ animals illustrate the altered distribution of SVs in the presynaptic terminals of the diaphragm muscle after electrical stimulation (Figure 2– F1–F4 – asterisks represent areas depleted of SVs near the plasma membrane).

**Figure 3:**
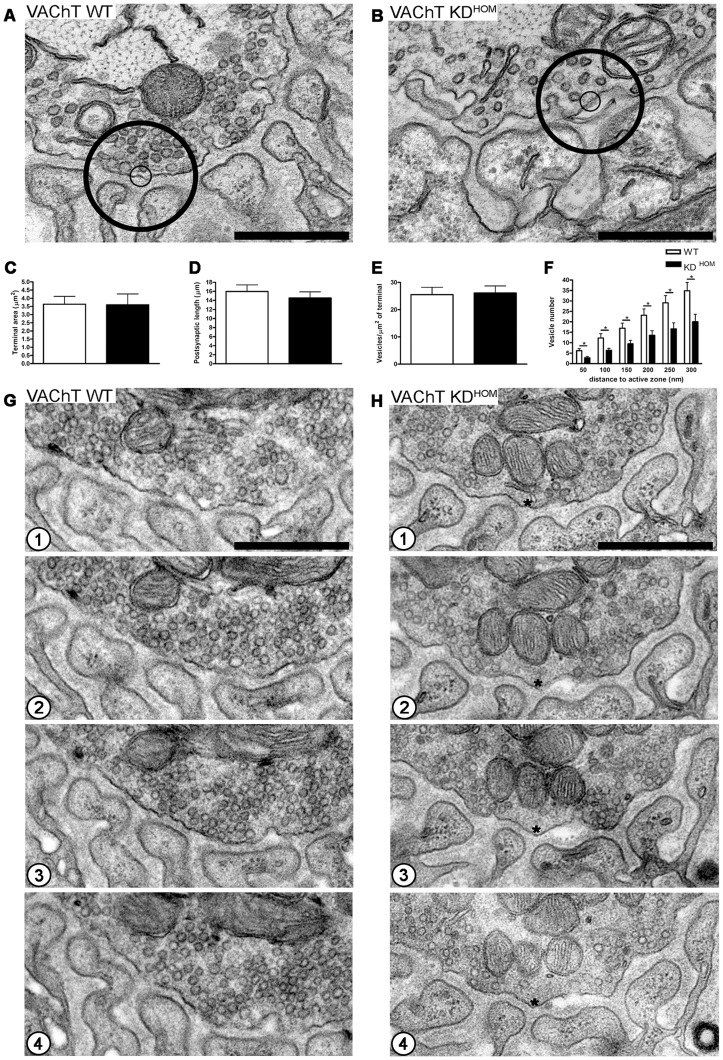
VAChT KD^HOM^ NMJs have normal morphology but altered SVs distribution in the absence of stimulus. A and B– Representative images of nerve terminal profile from VAChT WT and VAChT KD^HOM^ mice in the absence of stimulation showed a altered SVs distribution from the active zone within the circles: 50 and 300 nm from the membrane, small and big circles respectively. Scale bar  = 500 nm. Magnification 50.000x. C– Graph showing the area of the presynaptic terminals in μm^2^. D– Graph comparing the total postsynaptic membrane lenght (μm). E – Graph of the ratio SVs/area of presynaptic terminal in μm^2^. F– Graph showing the average number of SVs located at different distances from the presynaptic active zones. G and H– Four serial sections of the profile of NMJs of VAChT WT (G1–G4) and VAChT KD^HOM^ (H1–H4) mice showing the altered SVs distribution in the active zone (* represent depletion areas of SVs) of motor terminals of VAChT KD^HOM^ in the absence of stimulus. Scale bar  = 500 nm. Magnification 50.000x. (n = 5 individual animals per genotype. * p<0.05).

We also looked at the ultrastructure of motor endplates from the diaphragm of VAChT KD^HOM^ and WT mice in absence of stimulation. We found that the NMJs of VAChT KD^HOM^ and WT mice presented a very similar morphology, regarding terminal area, postsynaptic length and total number of SVs (Figure 3A and 3B- small and big circles standing for synaptic vesicles located 50 and 300 nm from the plasma membrane respectively). Morphometric analysis showed that there was no difference in the surface area of nerve endings (cross section area of nerve terminals) comparing VAChT WT (3.635±0.4854 μm^2^) and VAChT KD^HOM^ mice (3.601±0.6639 μm^2^) (Figure 3C – p>0.05; unpaired Student's *t*-test; 25 nerve terminals per genotype; n = 5 mice per condition). We also measured the length of the postsynaptic junctional folds considering possible compensatory changes in muscle cell due to the cholinergic deficit, but no differences were observed between genotypes (Figure 3D – WT  = 15.96±1.458 μm [mean ± SEM]; KD^HOM^  = 14.51±1.377 μm; p>0.05; unpaired Student's *t*-test; 25 nerve terminals profile per genotype; n = 5 mice per genotype).

Considering that VGLUT1 KO mice exhibit a reduction in the number of SVs in non-stimulated glutamatergic nerve terminals [28], we asked whether the decreased VAChT levels could have a similar effect in the number of SVs in cholinergic motor terminals. However, we observed no difference in the total number of SVs/μm^2^ of terminal between VAChT WT (25.0±3.0 SVs [mean ± SEM]) and VAChT KD^HOM^ (26.0±2.0 SVs) in the absence of stimulation (Figure 3E – p>0.05; unpaired Student's *t*-test; 25 nerve terminal profiles per genotype; n = 5 mice per genotype).

However, quantitative analysis showed an altered distribution of SVs located at different distances from the presynaptic active zone in VAChT KD^HOM^ when compared to VAChT WT mice in the absence of stimulation [Figure 3F –50 nm: WT  = 6.0 SVs (mean), KD^HOM^  = 3.0 SVs; 100 nm: WT  = 12.0 SVs, KD^HOM^  = 6.0 SVs; 150 nm: WT  = 17.0 SVs, KD^HOM^  = 9.0 SVs; 200 nm: WT  = 23.0 SVs, KD^HOM^  = 14.0 SVs; 250 nm: WT  = 29.0 SVs, KD^HOM^  = 17.0 SVs; 300 nm: WT  = 35.0 SVs, KD^HOM^  = 20.0 SVs; p<0.05, unpaired Student's *t*-test; (25 nerve terminals profiles per genotype; n = 5 mice per genotype)]. Figures 3G and 3H show four serial sections (50 nm thick) of unstimulated NMJs of VAChT WT (G1 – G4) and VAChT KD^HOM^ (H1–H4) mice, respectively, which allows a more accurate monitoring of the distribution of SVs in motor terminals. Altered distribution of SVs near active zones in nerve terminals of VAChT KD^HOM^ does not account for a change in the total number of vesicles (Figure 1E), probably because the SVs near active zones represent only a small fraction of the total number (about 500,000) of vesicles present in motor terminals of vertebrates [29].

**Figure 4:**
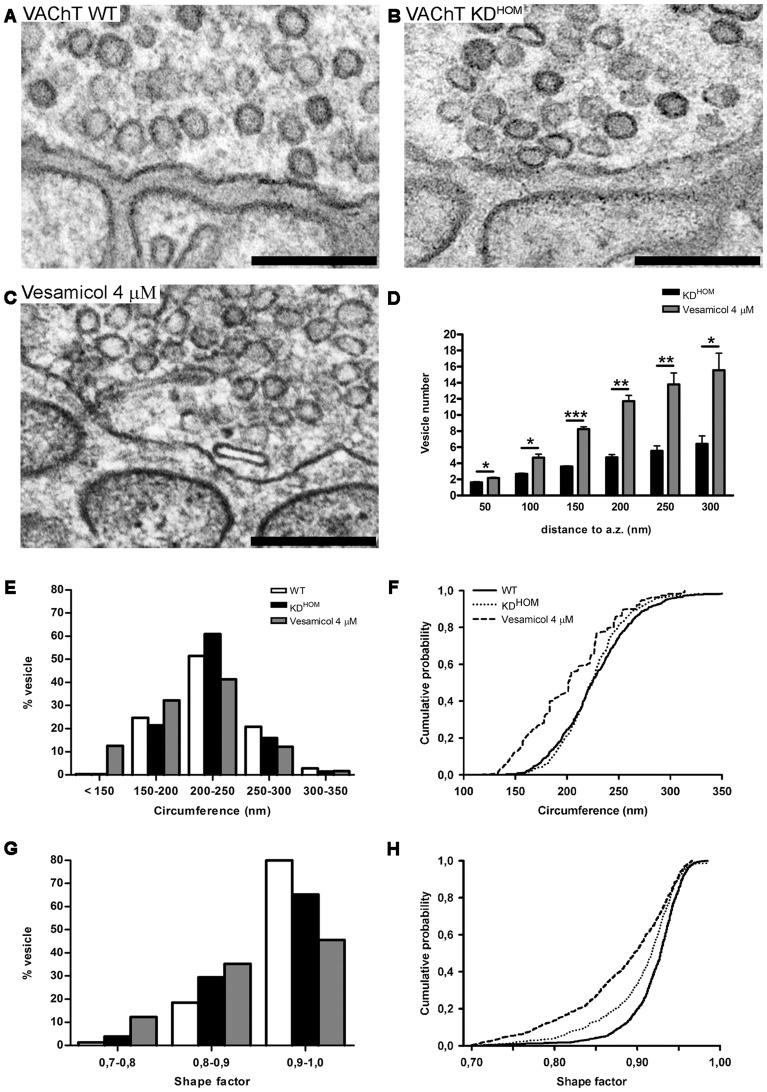
SVs morphology in nerve terminal from VAChT KD^HOM^ mice is influenced by neurotransmitter content. A and B – Representative images of nerve terminal profile from VAChT WT and VAChT KD^HOM^ mice after electrical stimulation (20 Hz for 5 minutes). Scale bar  = 500 nm. Magnification 50.000x. C– Representative image of nerve terminal profile from diaphragm muscle of WT mice after treatment with (±)-vesamicol (4 μM) during electrical stimulation (3Hz/20 min). Scale bar  = 500 nm. Magnification 50.000x. D– Graph showing the average number of SVs located at different distances from the presynaptic active zones (n = 3 individual animals for condition. * p<0.05, ** p<0.01; *** p<0.0001). E– Frequency histogram of SVs circumference measured from sections of NMJs from diaphragm of VAChT WT and VAChT KD^HOM^ mice after electrical stimulation and WT mice after treatment with (±)-vesamicol. F– Cumulative probability plot of the data in (E). (n = 3 individual animals per experimental condition). G– Frequency histogram of SVs shape VAChT WT and VAChT KD^HOM^ mice after electrical stimulation and WT mice after treatment with (±)-vesamicol. H– Cumulative probability plot of the data in (G). (n = 3 individual animals per experimental condition).

The size and shape of SVs and specialized secretory granules can be influenced by changes in neurotransmitter transporter expression or by the amount of transmitter stored. For instance, overexpression or reduced expression of VGLUT in *Drosophila* NMJs determine an increase or decrease in the diameter of SVs, respectively [30], [31]. Increased vesicular loading is coupled with an increase in specialized secretory vesicle volume [32], [33]. Additionally, the morphology of SVs also seem to correlate with neurotransmitter filling [8], [34]. Consistent with these findings, in the present work we observed that NMJs of VAChT KD^HOM^ mice show numerous vesicles with irregular morphology (flattened and elliptical) (Figure 4). To test whether the change in shape of SVs in motor terminals of VAChT KD^HOM^ mice occurs due to a reduction in ACh quantal content, we compared the circumference and shape of SVs of motor terminals from VAChT WT (Figure 4A), VAChT KD^HOM^ mice (Figure 4B) and WT mice treated with (±)-vesamicol (Figure 4C), a VAChT blocker [35], [36], [37]. Quantitative analysis show a similar total number of SVs in nerve terminals of VAChT KD^HOM^, VAChT WT (non-treated) and WT treated with (±)-vesamicol (not shown).

Additionally, we analyzed the distribution of SVs in motor terminals of VAChT KD^HOM^ (non-treated) and found a altered distribution of SVs located at different distances from presynaptic active zone when compared with the WT treated with (±)-vesamicol [Figure 4D – 50 nm: KD^HOM^  = 1.0 SVs (mean), Vesamicol  = 2.0 SVs; 100 nm: KD^HOM^  = 3.0 SVs, Vesamicol  = 5.0 SVs; 150 nm: KD^HOM^  = 4.0 SVs, Vesamicol  = 8.0 SVs; 200 nm: KD^HOM^  = 5.0 SVs, Vesamicol  = 12.0 SVs; 250 nm: KD^HOM^  = 6.0 SVs, Vesamicol  = 14.0 SVs; 300 : KD^HOM^  = 6.0 SVs, Vesamicol  = 16.0 SVs; p<0.05, unpaired Student's *t*-test; we analyzed 15 nerve terminals profiles per genotype; n = 3 mice per condition]. However, we observed that nerve terminals from VAChT KD^HOM^ exhibited SVs slightly smaller (224.0±1.0 nm) than those from VAChT WT (226.0±1.0 nm) (p<0.05; Kolmogorov-Smirnov test). We also observed that nerve terminals from WT treated with (±)-vesamicol presented even smaller SVs (203±2.0 nm) compared to VAChT KD^HOM^ and VAChT WT mice (p<0.0001; Kolmogorov-Smirnov test. Figure 4E and 4F –712 vesicles for WT and KD^HOM^ and 724 vesicles for vesamicol from 15 nerve terminal profiles for each experimental condition; n = 3 mice per condition). Furthermore, NMJs from both VAChT KD^HOM^ non-treated and WT mice treated with (±)-vesamicol showed a reduced number of SVs with spherical shape when compared with VAChT WT (non-treated) (p<0.0001; Kolmogorov-Smirnov test. Figure 4G and H –1104 vesicles for WT and KD^HOM^ and 1193 vesicles for vesamicol from 15 nerve terminal profiles for each experimental condition; n = 3 mice per experimental condition). These results suggest that the distribution and morphology of SVs in motor terminals from diaphragm NMJ of VAChT KD^HOM^ mice may be related to level of VAChT expression and ACh storage, respectively.

## Discussion

In this study, we investigated the impact of reduced expression of VAChT on the morphology of NMJs from the diaphragm muscle of VAChT KD^HOM^ adult mice. Using transmission electron microscopy we found that the synaptic elements of NMJs exhibited normal overall morphology concerning presynaptic terminals size, total number of SVs per terminal and postsynaptic membrane length, when compared with VAChT WT. Considering that ACh coordinates synaptic maturation [1], [3], [38], [39], our results suggest that reduced expression of VAChT ensures a minimal level of ACh release which is sufficient to maintain the development and normal formation of neuromuscular synapses in VAChT KD^HOM^ mice. Differently, VAChT^del/del^ or ChAT KO mice exhibit abnormal development of NMJs, showing increase in motoneurons and nerve terminals number, dilated motor endplates, profusion of ACh receptors in the proximity of nerve terminals, multiple synaptic sites on individual myotubes; hyperinnervation of individual synaptic sites and decreased number of junctional folds in the postsynaptic membrane [1], [2], [3].

A new finding of this study relates to our results using hypertonic sucrose to stimulate SV recycling from the RRP in motor nerve terminals from diaphragm of mice with cholinergic deficit. We found that VAChT KD^HOM^ mice exhibit reduced MEEP frequency and amplitude during hypertonic stimulation. Furthermore, we observed a reduction in FM1-43fx staining in these mice, compatible with the reduction in the total number of SVs revealed by ultrastructural analysis when compared to WT. Hypertonic extracellular solution increases MEPP frequency at the vertebrate NMJ [23], [24], [25], [26]. Although the mechanism for such enhancement is unknown, there are evidences suggesting that this stimulus does not require Ca^2+^ influx or release from internal stores and consists of a calcium-independent neurotransmitter release that mobilizes specifically the RRP [22], [27]. Therefore, our results suggest that, at least to hypertonic stimulation, the reduction in the MEPPs frequency does not occur only by competition between empty and filled vesicles [10], but also by considerable defect of SVs recycling from RRP.

The NMJ of vertebrates has a total vesicle pool of about 500,000 vesicles [29], which are divided into three pools showing distinct functional properties: the readily releasable pool (RRP), the recycling pool (RP) and the resting pool (R_t_P), according to the proposal for unifying terminology [40]. Aside from differences in spatial location, no other ultrastructural features clearly distinguish the SVs pools within a presynaptic terminal [29], [40]. Thus, subtle changes of SV distribution in motor terminals of VAChT KD^HOM^ would not be perceived during FM1-43 staining when considering the existence of such a large total pool. However, our ultrastructural data show altered SV distribution near active zones in hypertonically stimulated (Figure 1), electrically stimulated (Figure 2) and non-stimulated nerve terminals (Figure 3), suggesting a defect in vesicle mobilization in VAChT KD^HOM^ mice compared to WT. An elegant study performed in primary cultures of neonatal rat hippocampal neurons [41] suggested that SVs undergo alterations, or maturation processes that result in the reduction of their mobility and in their clustering into a preexisting pool. Interestingly, our data shows that synaptic vesicle distribution near the active zone in vesamicol treated WT mice differs from VAChT KD^HOM^ (Figures 4D). Based on this and the afore mentioned work in hippocampal neurons [41], we suggest that a change in the number of copies of VAChT per synaptic vesicle in VAChT KD^HOM^, may signal an immature state of cholinergic SVs and make them less mobile early, resulting in reduction in the clustering of SVs in individual pools and reduced interconversion of vesicles between pools [29], [40], [42].

Another possibility to explain the change in the SVs distribution in motor nerve terminals of VAChT KD^HOM^ mice could lie in the fact that changes in VAChT expression may impair the expression of proteins that regulate vesicle mobility and thereby impair the formation of vesicular pools or result in dispersion of vesicles. Some studies have shown a correlated expression between proteins involved with SVs mobility and vesicular neurotransmitter transporter from the central nervous system, especially to VGLUT-1, VGLUT-2 and VGAT [28], [43], but not VAChT [43]. However, it would be reasonable that presynaptic proteins could regulate the mobility of SVs in motor terminals of VAChT KD^HOM^, through the interaction with the VAChT. Future studies could focus on the mechanisms of interaction between VAChT and other presynaptic protein and the consequences of reduced expression of this transporter for the formation of SVs pools.

Another important finding of this work relates to the observed alteration in morphology of SVs from NMJ of VAChT KD^HOM^ mice. Considering that VAChT KD^HOM^ animals have a reduction in the number of copies of the transporter in the SVs membrane and that they exhibit reduced quantal ACh content [10], we hypothesized that the change in morphology of SVs is a consequence of the reduced filling with ACh. To test this hypothesis we compared circumference and shape of SVs from NMJ of VAChT KD^HOM^ mice and WT treated with vesamicol, a VAChT blocker [35], [36], [37]. Ultrastructural analysis revealed that the pharmacological inhibition of VAChT also changes the morphology of SVs.

The relationship between SVs size and changes in quantal acethylcholine content has been investigated specially at cholinergic nerve terminal from the frog NMJ [8]. At the NMJ cholinergic SV recycling continued to occur in nerve terminals stimulated in the presence of vesamicol, showing that transport of ACh into recycled vesicles is not a requisite for repeated SV cycle [44]. Experiments using hypertonic gluconate and aspartate solution to increase quantal size showed an increase in the size of MEPPs that was not accompanied by changes in SV size [8]. In addition, vesicle size was not substantially decreased when the quantal content was reduced by treatment with hemicholinium (inhibitor of choline uptake) or NH_4_^+^ (which diminishes the proton gradient for ACh uptake into the vesicles). However, treatment with vesamicol induced a decrease in vesicle size [8], which agrees with our findings from mice with reduced VAChT expression and treated with vesamicol described in Figure 4. Interestingly, previous work suggested that vesamicol may be altering vesicle size by a mechanism other than inhibiting VAChT [8], but our data showing changes in circumference and shape in VAChT KD^HOM^ and vesamicol treated nerve terminals indicate that this might not be the case at least in the mice NMJ.

VAChT is a transmembrane protein that uses the electrochemical gradient generated by a V-type proton ATPase to accumulate ACh in SVs [4], [7], [45], [46]. Therefore, a change in the VAChT activity could impact on proton exchange, changing tonicity and inducing morphological changes in SVs. Indeed, it has been recently reported [47] that aldehyde fixation induces flattening of SVs in hippocampal synapses of VGLUT1^−/−^ mice due to an alteration in the tonicity of excitatory SVs. We therefore suggest that in cholinergic vesicles the normal expression and activity of VAChT are also important for maintaining tonicity and morphology of SVs in nerve terminals from diaphragm NMJ.

Even though our results suggest that ACh content interferes with the morphology of SVs we cannot rule out the possibility that the reduced VAChT protein levels or activity in our experimental model may also affect vesicle shape. Removal of plasma membrane components, such as cholesterol, does not alter the SVs shape, although considerably alters circumference in frog NMJ [48]. Furthermore, overexpression or reduced expression of VGLUT in *Drosophila* NMJ induces an increase or decrease in the diameter of SVs, respectively [30], [31]. Additionally, morphological aspect of SVs may also be defined after clathrin-mediated endocytosis [49], [50], [51]. Considering that VAChT interacts with clathrin adaptors [52], [53], [54], reduced expression of this transporter could compromise the number of sites necessary for proper connection between them. Therefore, it is possible that changes in shape of SVs from motor nerve terminals of VAChT KD^HOM^ mice may also be related to a defect in modeling during endocytosis. One intriguing possibility is that these changes in SVs circumference and shape that we detect in the absence of VAChT may be the reason for the altered recycling of SVs in the RRP that we observed in these mutant mice.

In conclusion, our data show that decreased VAChT expression play a role in recycling and mobilization of specific pools of SV in NMJ. We suggest that quantal ACh content and reduced VAChT protein levels or activity are important to define the morphology and distribution of SVs and the recycling of the RRP. Our results also suggest that functional alterations caused by VAChT deficiency [9], [55], [56] may involve multiple mechanisms, including a decreased in neurotransmitter storage in addition to deficits in the recycling and mobilization of the RRP. Future studies will be needed to clarify the relation between expression of VAChT and regulation of SVs mobility in neuromuscular synapses.
